# Humidity Effect on Dynamic Electromechanical Properties of Polyacrylic Dielectric Elastomer: An Experimental Study

**DOI:** 10.3390/polym13050784

**Published:** 2021-03-04

**Authors:** Yuchen Zuo, Yuxi Ding, Junshi Zhang, Mingliang Zhu, Lei Liu, Jianwen Zhao

**Affiliations:** 1 School of Aeronautics, Northwestern Polytechnical University, Xi’an 710072, China; zycneu@163.com; 2 School of Mechanical Engineering & Automation, Northeastern University, Shenyang 110819, China; 3 School of Mechanical and Precision Instrument Engineering, Xi’an University of Technology, Xi’an 710048, China; yuxiding62@gmail.com (Y.D.); zzxhhi202103@163.com (M.Z.); aist456@163.com (L.L.); 4 School of Mechanical Engineering, Harbin Institute of Technology, Weihai 264209, China; zhaojianwen@hit.edu.cn

**Keywords:** dielectric elastomer, humidity effect, dynamic response, viscoelastic creeping

## Abstract

In this research, by utilizing the Very-High-Bond (VHB) 4905 elastomer, we carry out an experimental examination on the humidity effect on dynamic electromechanical performances of dielectric elastomers, including the dynamic response and viscoelastic creeping. Firstly, we experimentally analyze effects of the pre-stretch, peak voltage, waveform and frequency of the dynamic response of VHB 4905 elastomer under several ambient humidities. In general, the amplitude of dynamic deformation gradually adds up with the increasing humidity. Besides, it is found that the amplitude affected by different parameters shows diverse sensitivity to humidity. Subsequently, effect of humidity on the viscoelastic creeping of VHB 4905 is explored. The results demonstrate that, subject to different ambient humidities, the viscoelastic creeping under Alternating Current (AC) voltage is similar to that under Direct Current (DC) voltage. Furthermore, the equilibrium position of dynamic viscoelastic creep enlarges gradually with the humidity, regardless of voltage waveforms. For the dielectric elastomer with a pre-stretch ratio of 3, when the humidity increases from 20% to 80%, the increase of average equilibrium position of dynamic viscoelastic creep is larger than 1599%.

## 1. Introduction

Dielectric elastomers (DEs), a category of soft electro-active polymers, have gained much attention recently because of their excellent properties, such as high energy density, large deformability fast response and so on [1,2,3,4,5,6]. DEs have demonstrated significant potential in applications such as soft actuators, bionic robots and energy generators [7,8,9,10,11,12,13]. The majority of DE materials belong to macromolecular polymers, which are very sensitive to the external environment, such as the temperature and humidity. Therefore, the electromechanical deformation and stability of DEs is greatly affected, when exposed to the external environment. The researchers have found that the temperature effect plays an important role in determining the static electromechanical actuation of DEs [14,15,16,17,18,19,20]. During recent years, some scholars also have committed to investigations about humidity effects on the static electromechanical actuation of DEs [21,22,23,24,25,26]. In 2016, Chen et al. [21] carried out research on the effect of humidity on the electrical breakdown strengths of VHB 4905 membranes. Then, Fasolt et al. [22] experimentally presented an investigation in which the effect of humidity on the breakdown field of silicone DE films was tested. Subsequently, by utilizing the silicone DE membranes, Albuquerque et al. [23] experimentally reported the breakdown strength with consideration of the humidity effect. In our recent researches [24,25,26], we investigated the humidity effect on the static actuation performances of VHB 4910, both theoretically and experimentally.

Nevertheless, the DEs are more widely used as dynamic actuators, and some researchers have analyzed the dynamic performances of DEs under constant environmental conditions. For example, Sheng et al. [27] proposed a free energy model to investigate the dynamics of a DE membrane undergoing in-plane deformation. Chen et al. [28] derived a high nonlinearity motion equation and presented the dynamic response of the DE balloon actuator subject to a combination of pressure and periodic voltage. Lv et al. [29] developed a theoretical model incorporating the stiffening and damping effect, investigated the dynamic performance of a DE balloon subject to electromechanical coupling loads. Kashyap et al. [30] developed a dynamic model to describe the dynamic response of a DE actuator for different values of viscoelasticity and anisotropy parameters.

Although the above experiments have legitimately explored the humidity effects on static electromechanical properties of DEs and the dynamic electromechanical properties of DEs under constant environment conditions. However, too little work has been devoted to determination of the humidity effect on dynamic electromechanical properties of DEs. When the dynamic DE actuators in the moisture environment (such as the underwater DE electronic fish [8,9]), the electromechanical properties will be largely affected by the working humidity. Therefore, the humidity effects on the dynamic electromechanical performance of DEs arguably are significant questions to be addressed. Accordingly, in this manuscript, we experimentally studied the humidity effects on the dynamic electromechanical properties of DEs, mainly focusing on the dynamic response and viscoelastic creeping. This research offers an insight into the VHB-based DE actuator for device design, modeling and control strategies in varying ambient humidity.

## 2. Experimental

### 2.1. Experimental Procedures and Setups

**Figure 1:**
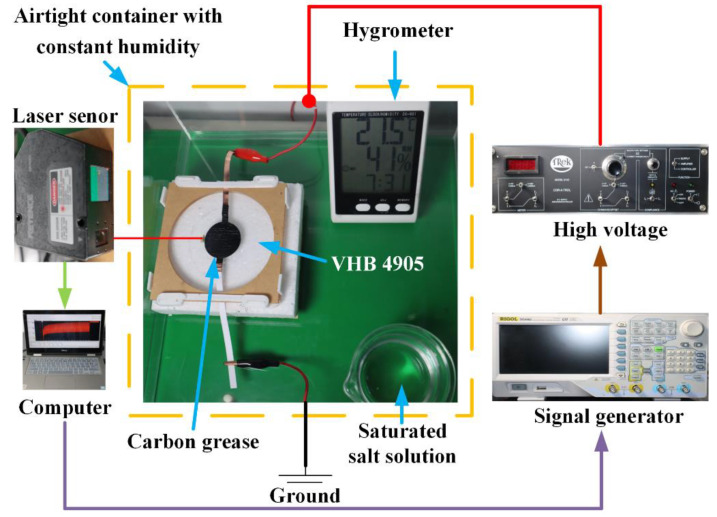
Experimental procedures of VHB 4905 film under different humidity levels.

**Figure 2:**
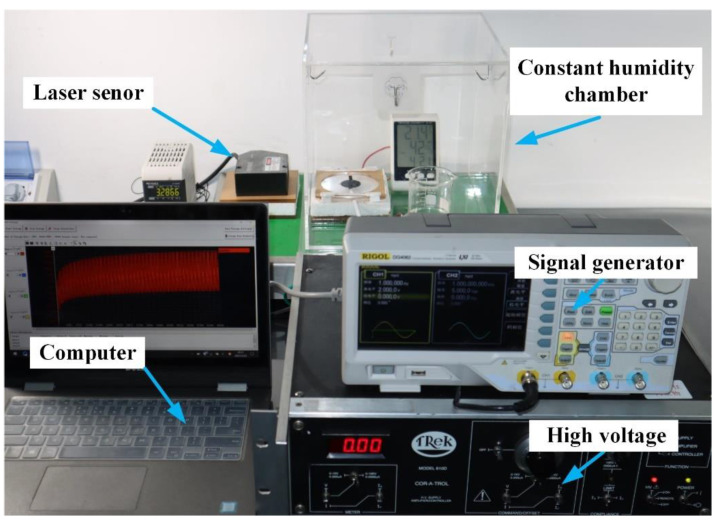
Experimental setups for electromechanical measurement of VHB 4905 film under different humidity levels.

In this article, under room temperature (20 °C), we investigate the humidity effect on the dynamic electromechanical properties of DEs, by applying an AC voltage. The electromechanical testing procedures are illustrated in Figure 1, and the experimental setups are demonstrated in Figure 2. VHB 4905 (3M Company, Sao Paulo, MN, USA) with an original thickness of 0.5 mm is used as the DE material because of its quick response and high deformability. VHB 4905 membrane is, first, equal-biaxially stretched with different pre-stretch ratios. Then we clamp the pre-stretched films by a pair of annular frames to maintain the prescribed pre-stretch ratio. The carbon grease electrodes (no. 846, MG Chemicals, Burlington, ON, Canada) are coated in the center area of both surfaces, forming a circular configuration with a diameter of 30 mm. Each VHB sample is placed in the airtight container for at least 1 h to make sure the VHB 4905 films are fully compatible with the environment. In order to eliminate the errors, five samples are fabricated for each measurement. The various humidity levels can be approximately concerted by different saturated salt solutions, including potassium acetate (CH_3_COOK), potassium carbonate (K_2_CO_3_), sodium bromide (NaBr) and potassium bromide (KBr), which are placed in the airtight container [24]. A high voltage supplier (Model 610D, Trek, New York, NY, USA) is utilized to produce the high voltage for actuation of VHB 4905 elastomer, and voltage signal is generated by a signal generator (DG4062, Rigol, Suzhou, China). A laser sensor (LK-G80, Kenyence, Osaka, Japan) is used to measure the electromechanical displacement of the VHB 4905 film, by attaching a lightweight marker on the edge of the electroactive area. Finally, the experimental data are collected by a DAQ card (USB6003, Ni, Austin, TX, USA) and inputted into the computer to obtain the results.

### 2.2. Experimental Method

We vary the peak voltage (the peak voltage means the peak value of applied AC voltage), waveform, and frequency of applied voltage and pre-stretch ratio of VHB 4905 film under four humidity levels (20%, 40%, 60% and 80%) in our experiments. Under different ambient humidities, we tested three pre-stretch ratios, three waveforms and four frequencies under the same nominal electric field. Furthermore, for each pre-stretch ratio (2, 3, 4), we also tested four different peak voltages. Table 1 shows the experimental parameters, in which three different waveforms, including sinusoidal, triangle and saw-tooth, are utilized.

## 3. Results and Discussion

### 3.1. Dynamic Response under Different Humidities

**Figure 3:**
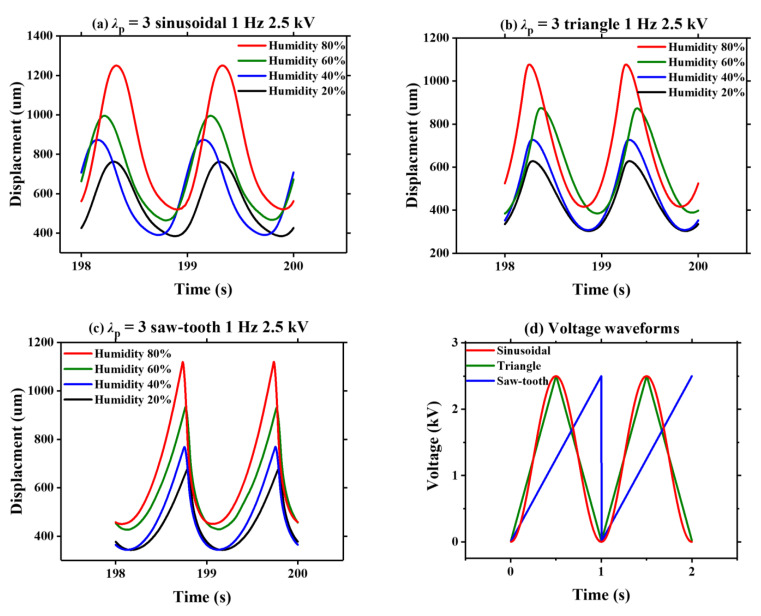
Displacements induced by three voltage waveforms under different humidity levels (20%, 40%, 60% and 80%): (**a**) sinusoidal signal, (**b**) triangle signal and (**c**) saw-tooth signal; (**d**) three voltage waveforms with a frequency of 1 Hz and peak voltage of 2.5 kV.

The voltage-induced dynamic deformation of VHB 4905 film under different ambient humidities is presented in Figure 3. As mentioned previously, three voltage waveforms with a frequency of 1 Hz and peak voltage of 2.5 kV are used (Figure 3d). With a prescribed pre-stretch radio *λ*_p_ = 3, Figure 3a–c show the corresponding dynamic displacement of VHB 4905, under four humidity levels (20%, 40%, 60% and 80%). Due to viscoelastic creeping, the timescale of 198–200 s is selected to present the stable dynamic displacement. It is demonstrated that the dynamic displacement of VHB 4905 gradually increases with the increasing humidity under the actuation of a random voltage waveform. Furthermore, the peak of dynamic response of the DE membrane under a sinusoidal voltage is the broadest, and there is a sharp pinnacle when a saw-tooth voltage is applied. Note that these responses are characterized by narrow peaks and broad valleys, mainly because of the nonlinear electrical loading. The amplitude was calculated by taking as half of the difference between the peak value and valley value of the dynamic response. As shown in Figure 3a–c, under different voltage waveforms, the amplitude of dynamic response of VHB 4905 enhances gradually as the ambient humidity increases. 

**Figure 4:**
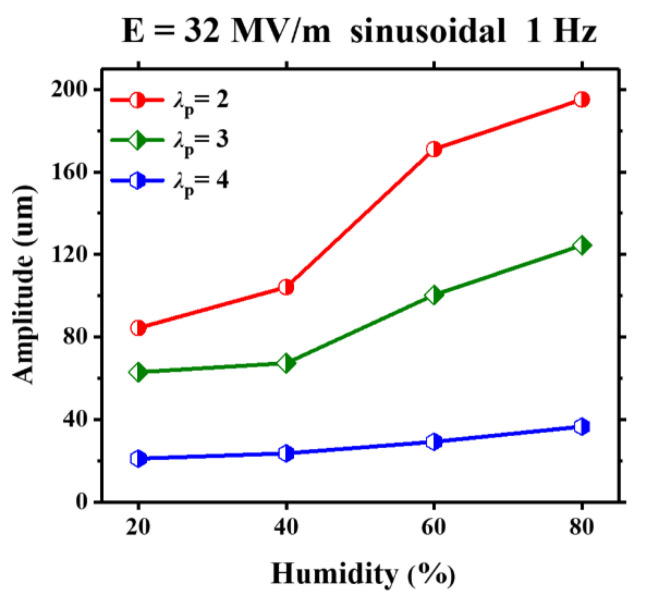
Subject to a sinusoidal voltage, amplitude of the VHB 4905 film under different pre-stretch ratios and ambient humidities.

For the VHB 4905 with three different equal-biaxial pre-stretch ratios (*λ*_p_ = 2, *λ*_p_ = 3 and *λ*_p_ = 4), Figure 4 presents the humidity effect on the amplitude of dynamic response under a sinusoidal voltage with a frequency of 1 Hz and a nominal electric field of 32 MV m ^−1^. It is noted that the amplitude increases gradually with the increasing humidity, especially when the humidity enlarges from 40% to 80%. Based on our previous research [25], the shear modulus of viscoelastic materials decreases gradually when the ambient humidity adds up. It is implied that the ambient humidity leads to the softening of the viscoelastic membrane. Under the same actuation, a softer film generates a larger electromechanical deformation. On the other hand, for a given value of ambient humidity, there is a reduction of the amplitude when the pre-stretch ratio enlarges from *λ*_p_ = 2 to *λ*_p_ = 4. This can be explained as follows: a large pre-stretch stiffens the VHB film and limits a large deformation.

**Figure 5:**
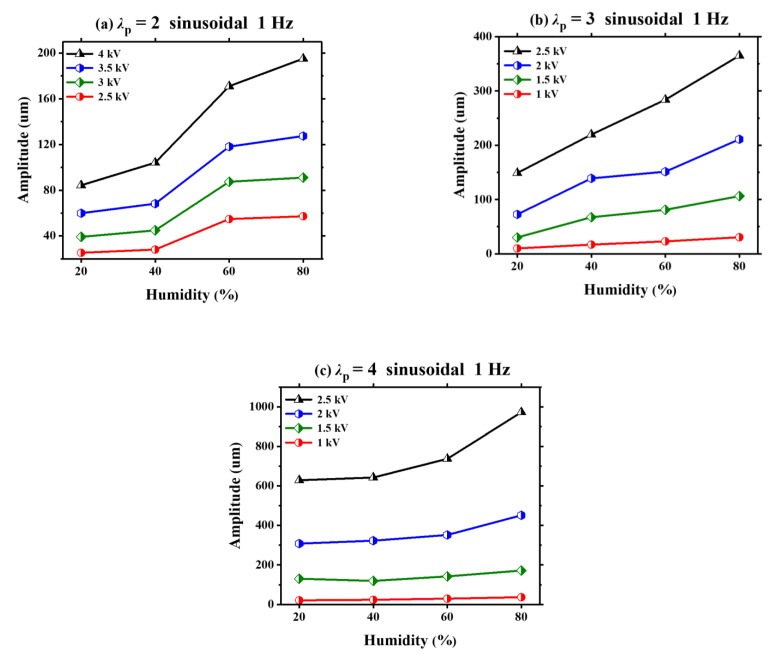
Under different pre-stretch ratios (*λ*_p_ = 2, *λ*_p_ = 3 and *λ*_p_ = 4), the humidity effect on the amplitude of VHB 4905 with different peak voltages.

Next, using a sinusoidal voltage, the humidity effect on the amplitude of VHB 4905 under three equal-biaxial pre-stretch ratios (*λ*_p_ = 2, *λ*_p_ = 3 and *λ*_p_ = 4) is investigated by using different peak voltages, which is displayed in Figure 5. In order to avoid the possible electrical breakdown and obtain the valid experimental data in this experimental measurement, we use 2.5 kV~4.5 kV as the range of peak voltage for pre-stretch ratio *λ*_p_ = 2 and 1 kV~2.5 kV as the range of peak voltage for pre-stretch ratios *λ*_p_ = 3 and *λ*_p_ = 4. It is found that for a given pre-stretch ratio, the amplitude of VHB 4905 increases with the increasing humidity for all different peak voltages, and the high peak voltage greatly augments the amplitude of VHB 4905 with the increase of humidity, indicating that high peak voltage causes the dynamic deformation to be more sensitive to the humidity. This phenomenon may be due to the nonlinear characteristics of DE membranes, which is surely affected by the varying humidity.

**Figure 6:**
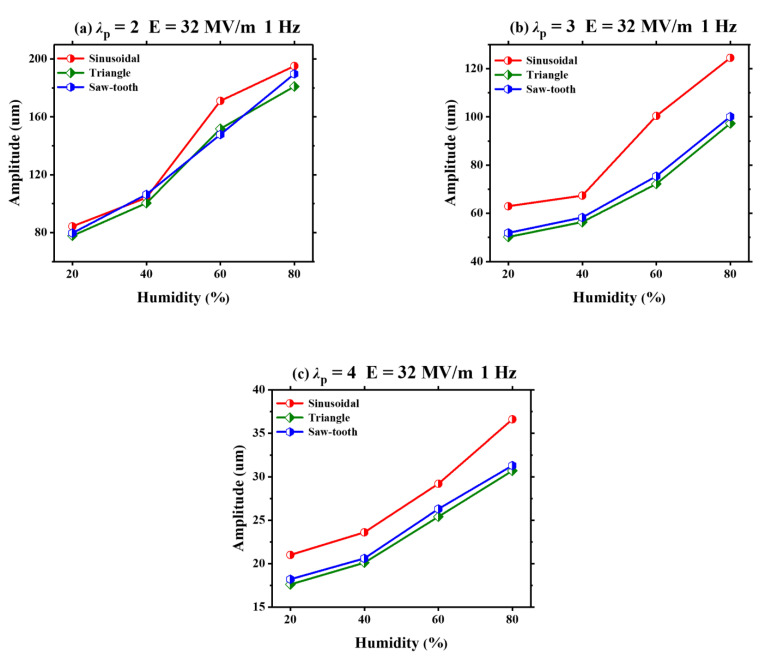
Under different pre-stretch ratios (*λ*_p_ = 2, *λ*_p_ = 3, and *λ*_p_ = 4), the humidity effect on the amplitude of the VHB 4905 film with different voltage waveforms (sinusoidal, triangle and saw-tooth).

Similarly, under three different pre-stretch ratios (*λ*_p_ = 2, *λ*_p_ = 3 and *λ*_p_ = 4), the humidity effect on the amplitude of VHB 4905 is investigated by using three voltage waveforms, which is displayed in Figure 6. Under a given pre-stretch ratio, the amplitude of VHB 4905 increases gradually with the increasing humidity for all different voltage waveforms. Furthermore, it is found that the sinusoidal voltage can generate a relatively larger amplitude compared with those generated by triangle and sawtooth voltages. On the other hand, regardless of the pre-stretch ratio, the sawtooth voltage generally generates a slightly higher amplitude than that generated by the triangle voltage. The reasons are given as follows. Firstly, despite all the three waveforms having equal peak values, the effective value of sinusoidal voltage is higher, and has more energy. Therefore, the induced amplitude is larger. In addition, although the triangle and sawtooth voltage theoretically have the same energy, the impossible instantaneous jump from +2.5 kV to 0 kV leads to a slightly large energy of sawtooth voltage and induces a slightly-large amplitude.

**Figure 7:**
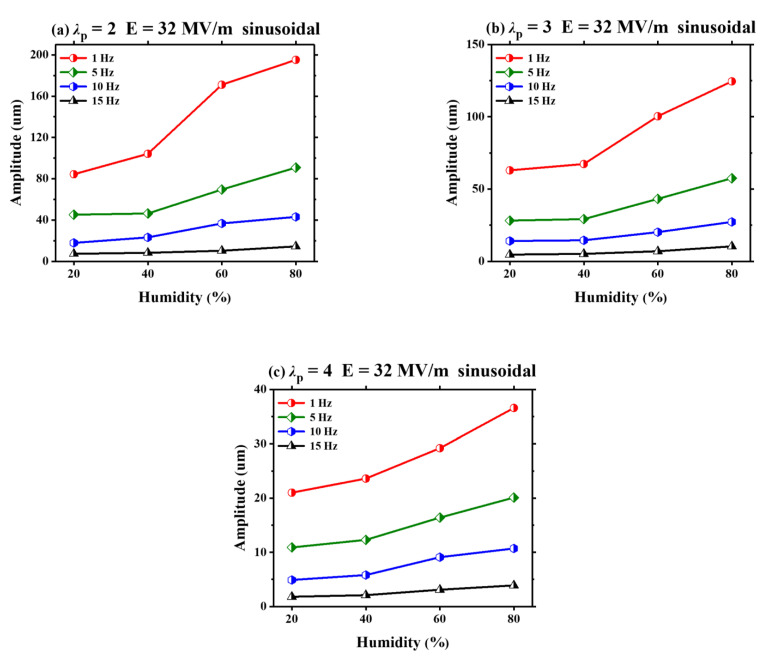
Under different pre-stretch ratios (*λ*_p_ = 2, *λ*_p_ = 3, *λ*_p_ = 4), the humidity effect on the amplitude of the VHB 4905 film with different frequencies (1Hz, 5Hz, 10Hz, 15Hz).

Finally, under three different pre-stretch ratios (*λ*_p_ = 2, *λ*_p_ = 3 and *λ*_p_ = 4), the humidity effect on the amplitude of VHB 4905 is investigated by using a sinusoidal voltage with four different frequencies, which is illustrated in Figure 7. Similar to Figure 5, under various humidity levels, the sinusoidal voltage with a low frequency generates a large amplitude. As the frequency increases, the amplitude drops sharply. When the frequency is up to 10 Hz, the produced deformation is extremely small. A similar phenomenon has been reported in the experiments of out-of-plane deformation [31]. Besides, for a given pre-stretch ratio, the amplitude adds up with the increasing humidity for all different frequencies, and a low frequency induces a rapid increase of the amplitude with the humidity.

### 3.2. Viscoelastic Creeping under Different Humidities

**Figure 8:**
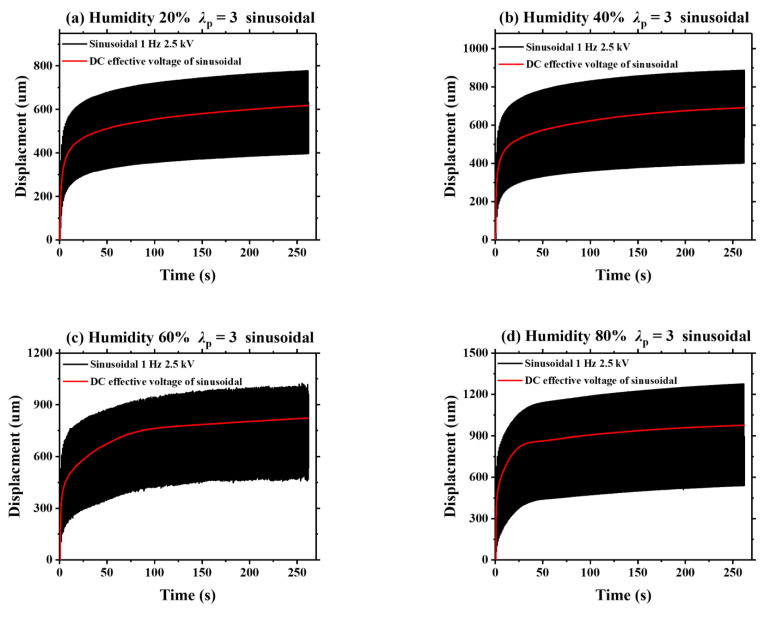
Comparison of the displacements between the effective DC signal and the sinusoidal signal under different ambient humidities.

**Figure 9:**
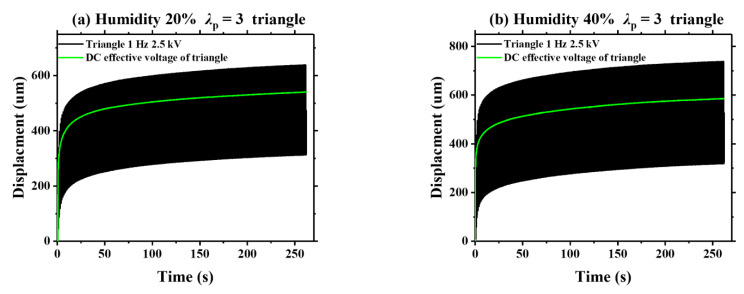

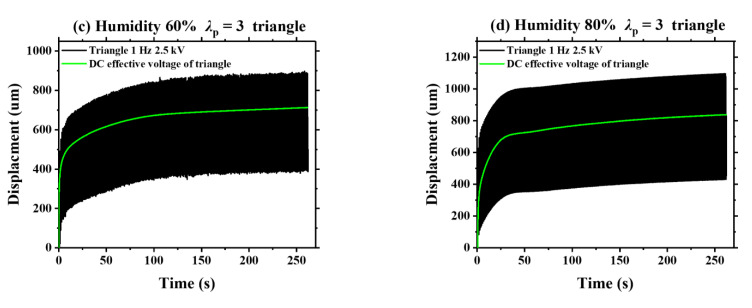
Comparison of the displacements between the effective DC signal and the triangle signal under different ambient humidities.

**Figure 10:**
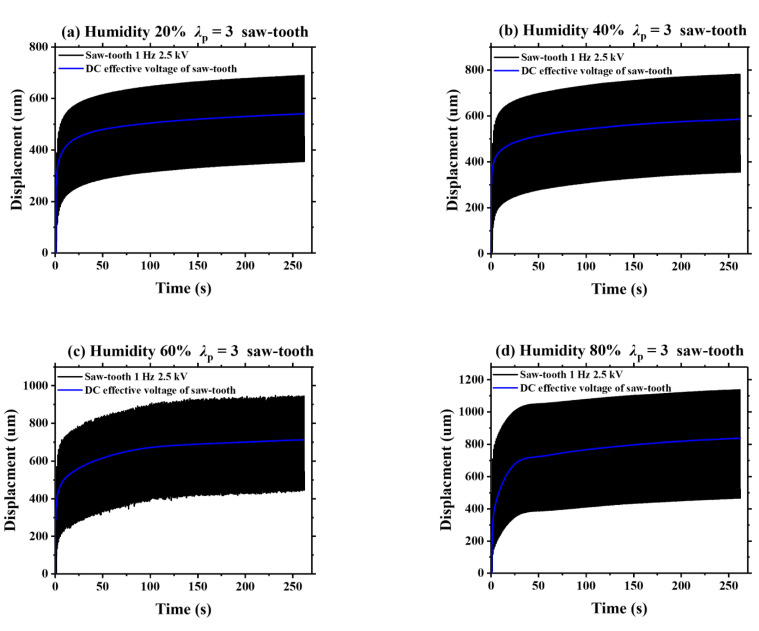
Comparison of the displacements between the effective DC signal and the saw-tooth signal under different ambient humidities.

As is known, viscoelasticity is an inherent property of DE materials. In this section, we detect the humidity effect on the viscoelastic creep of VHB 4905 elastomer. Figure 8 displays the dynamic viscoelastic creeping of VHB 4905 (*λ*_p_ = 3) when a sinusoidal voltage with a frequency of 1 Hz and a peak voltage of 2.5 kV is applied. Meanwhile, the effective DC voltage of the applied sinusoidal voltage is calculated, and the static deformation of the effective DC voltage is simultaneously measured. Under different ambient humidities, the increase of viscoelastic creep deformation for the VHB 4905 membrane under sinusoidal voltage is similar to that under the effective DC voltage. It is noted that the slopes of displacement-time curves are continuously declining, implying a gradually-weakened viscoelastic creep deformation of VHB 4905 elastomer. Based on these characteristics, it can be predicted that such creeping can be eliminated if the testing time is long enough. Similar results can be found when the triangle and sawtooth voltages are applied, which are shown in Figure 9 and Figure 10.

**Figure 11:**
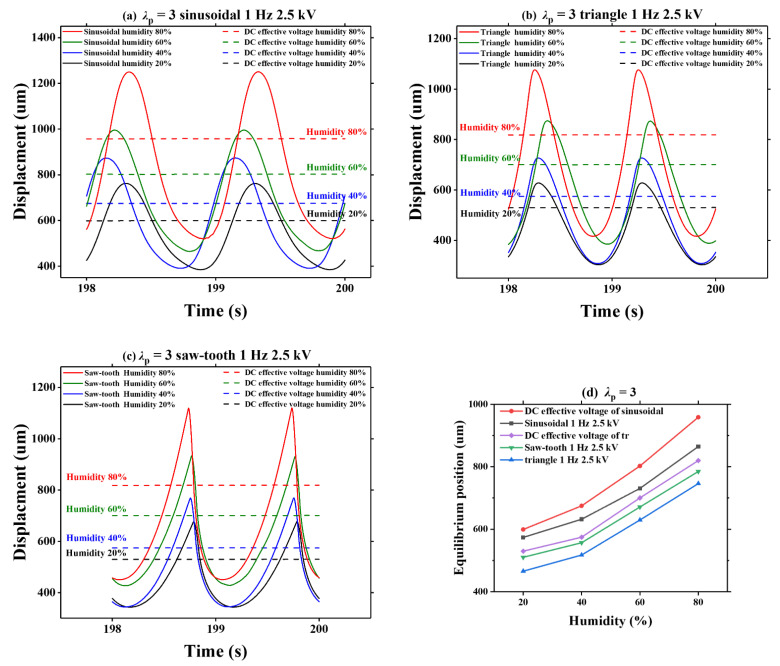
Under different voltage waveforms (sinusoidal, triangle, saw-tooth), experimental results on humidity effects on the viscoelastic creeping of the equilibrium position with the effective DC voltage and the AC voltage: (**a**) pre-stretch ratio of 3 and sinusoidal voltage; (**b**) pre-stretch ratio of 3 and triangle voltage; (**c**) pre-stretch ratio of 3 and saw-tooth voltage; (**d**) the equilibrium position of VHB 4905 under pre-stretch ratio of 3 and different voltage waveforms.

We use the equilibrium position to evaluate the degree of viscoelastic creeping within the timescale of 198–200 s. The equilibrium position is defined as half of the sum of the peak value and valley value of the dynamic deformation. Figure 11a–c present the dynamic displacement of VHB 4905 under different humidity levels, when the AC voltages and corresponding effective DC voltages are applied. Figure 11d summarizes the effect of humidity on the equilibrium position under three voltage waveforms and their corresponding effective DC voltage. Among them, since the triangle and saw-tooth voltages have the same effective value, we use “DC effective voltage of tr”, as shown in Figure 11d.

It can be seen, for each given voltage waveform, that the equilibrium position gradually increases with the increasing humidity, and the trend of increase is almost the same. From Table 2, we can find that when the humidity increases from 20% to 80%, the increase of average equilibrium position of dynamic viscoelastic creep is larger than 1599%. Similar to the amplitudes under various voltage waveforms, the humidity effect on equilibrium position is also less sensitive to voltage waveform. Moreover, it is noted that the sinusoidal voltage can produce a relatively higher equilibrium position compared with those produced by triangle and sawtooth voltages. On the other hand, the sawtooth voltage produces a slightly higher equilibrium position than that produced by the triangle voltage in most cases. This phenomenon is consistent with the amplitude of dynamic response, which may be caused by different energy of different voltage waveforms. In addition, the equilibrium position under the three AC voltages is slightly lower than that under the corresponding effective DC voltage, which is induced by the potential energy dissipation during dynamic vibration when the AC voltage is applied.

## 4. Conclusions

In this paper, we investigate the humidity effect on the dynamic electromechanical properties of VHB 4905 elastomer. Firstly, the effect of humidity on the dynamic response of VHB 4905 is explored. In general, the amplitude of dynamic deformation gradually increases with the increasing humidity. Among them, there are a variety of parameters that play distinct effects on the dynamic electromechanical performance of DE, such as pre-stretch, peak voltage, waveform and frequency. The amplitude is highly sensitive to the change of humidity at low pre-stretch ratio, high voltage peak value and low frequency. However, the amplitude under the random voltage waveform is less sensitive to humidity. 

In the following, the effect of humidity on the viscoelastic creeping of VHB 4905 is investigated. For different ambient humidities, the viscoelastic creeping is consistent when the DEs are under sinusoidal voltage, triangular voltage, sawtooth voltage and corresponding effective DC voltage. The experiment also analyzes the equilibrium position of viscoelastic creeping. For each given voltage waveform, the equilibrium position gradually rises with the increasing humidity. For the DE with a pre-stretch ratio of 3, when the humidity increases from 20% to 80%, the average equilibrium position of dynamic viscoelastic creep increases by 15.99 times. These investigations offer a strategy to comprehend the polyacrylic DEs for performance improvements and device explorations in some extremely moist environmental conditions. For future research, we hope to establish a dynamic model of the DE actuators incorporating the humidity effect.

## Figures and Tables

**Table 1:** Humidities, pre-stretch ratios, initial nominal electric strengths, peak voltages, frequencies and waveforms for electromechanical experiments.

Parameters	Value
Humidity (%)	20, 40, 60, 80
Pre-stretch ratio *λ*_p_	2, 3, 4
Peak value of the nominal *E* (MV m^−^^1^)	32
Peak voltage (kV)	2.5, 3, 3.5, 4(λ_p_ = 2); 1, 1.5, 2, 2.5 (*λ*_p_ = 3 and 4)
Frequency (Hz)	1, 5, 10, 15
Waveform	sinusoidal, triangle, saw-tooth

**Table 2:** The equilibrium position (um) under different voltage waveforms and different ambient humidities.

Humidity	20%	40%	60%	80%
Sin	573.6	632.0	730.8	864.8
DC effective voltage of sin	599.1	674.8	802.5	958.7
Saw-tooth	465.6	517.4	629.6	746.2
Triangle	510.1	556.7	671.2	784.9
DC effective voltage of tr	529.7	574.3	700.3	819.8

## Data Availability

The data presented in this study are available on request from the corresponding author.
